# Comparison of oxygen desaturation area-based methods in predicting cardiovascular disease-related mortality outcomes

**DOI:** 10.3389/fnetp.2026.1805587

**Published:** 2026-04-20

**Authors:** Siying He, Peter A. Cistulli, Philip de Chazal

**Affiliations:** 1 Charles Perkins Centre, Faculty of Engineering, Sydney University, Camperdown, NSW, Australia; 2 Charles Perkins Centre, Faculty of Medicine and Health, Sydney University, Camperdown, NSW, Australia; 3 Department of Respiratory and Sleep Medicine, Royal North Shore Hospital, St Leonards, NSW, Australia

**Keywords:** obstructive sleep apnea, cardiovascular disease, pulse oximetry, desaturation area, hypoxic burden, desaturation severity, respiratory event desaturation transient area, network physiology

## Abstract

**Study objectives:**

Desaturation area-based parameters derived from oximetry have emerged as novel predictors of cardiovascular disease mortality. Existing algorithms estimate the area under the oxygen desaturation curve but differ in computational aspects due to variations in baseline, sampling-window, and sleep event choice. These differences result in varying computational complexity and predictive performance. This study systematically characterizes published desaturation area-based algorithms to identify the most effective method for predicting cardiovascular disease-related (CVD) mortality and addressed the influence of computational discrepancy in the prediction.

**Methods and Results:**

This study utilized data from the Sleep Heart Health Study, including corresponding CVD mortality outcomes and covariates. A total of 4,483 participants (53.4% female; mean age: 64.32 years) were analyzed. Fifteen desaturation area methods were implemented based on variations of 3 published algorithms (hypoxic burden, desaturation severity, respiratory event desaturation transient area). The predictive performance of each method was assessed using Cox proportional hazards regression analysis, with adjustments for relevant covariates. A variation based on the hypoxic burden algorithm that used a record-specific baseline was the best-performing method for predicting CVD mortality outcomes. In the fully adjusted model, it demonstrated the strongest predictive performance, with a hazard ratio of 1.79 and a 95% confidence interval of 1.00–3.19 for predicting CVD mortality (p < 0.05).

**Conclusion:**

Computational discrepancies, particularly the choice of sleep event annotations, were found to have a substantial impact on the predictive ability of desaturation area–based parameters for CVD mortality. Among all evaluated methods, the approach based on hypoxic burden with a record-specific baseline demonstrated the strongest predictive performance.

## 1 Introduction

Obstructive sleep apnea (OSA) is a prevalent sleep disorder stemming from recurrent upper airway collapse during sleep. The immediate consequences of the obstructive breathing encompass intermittent hypoxia, cortical arousal, and sleep fragmentation, resulting in daytime sleepiness, metabolic complications, and adverse neurocognitive function (Jean-Louis et al., 2009). Cumulative hypoxic events contribute to the burden of nocturnal hypoxia, exacerbate sympathetic nervous system actions and vascular inflammation, thereby increasing the cardiovascular disease (CVD) risk, the leading cause of death worldwide (Baumert et al., 2020; Linz et al., 2015). OSA is highly prevalent in patients with CVD: 43%–73% of atrial fibrillation cases, 47%–76% of heart failure cases, and 30.5%–56% of coronary artery disease cases having OSA (Marshall et al., 2008; Altmann et al., 2012; Sin et al., 1999; Schäfer et al., 1999; Peker et al., 2002).

AHI is the standard measure of OSA severity and quantifies the number of apnea and hypopnea events per hour of sleep (Eiseman et al., 2012; Geovanini et al., 2018; Malhotra et al., 2021; Singh et al., 2020; Taranto-Montemurro et al., 2019). However, many studies suggest that AHI does not adequately represent the OSA–CVD association. It fails to capture factors such as respiratory event characteristics which exert critical impacts on the cardiovascular system (Azarbarzin et al., 2019; Butler et al., 2019; Punjabi, 2016). With the growing recognition of these limitations, oximetry-derived desaturation area–based parameters have been employed to analyze this relationship (Azarbarzin et al., 2019; Garvey et al., 2009; Mazzotti et al., 2020; He et al., 2023a).

**FIGURE 1:**
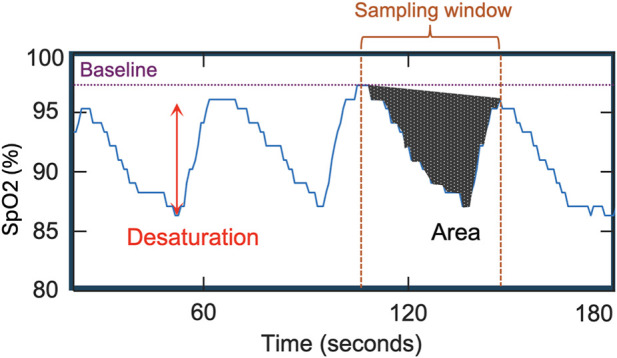
Schematic representation of desaturation area quantification from the SpO2 signal. Desaturation is defined as the reduction in SpO2 from the established baseline (red arrow). The desaturation area corresponds to the cumulative area associated with the desaturation event within a predefined sampling window. The sampling window (brown dashed lines) and baseline (purple dotted line) jointly determine the calculated desaturation area for a single event (shaded region).

**FIGURE 2:**
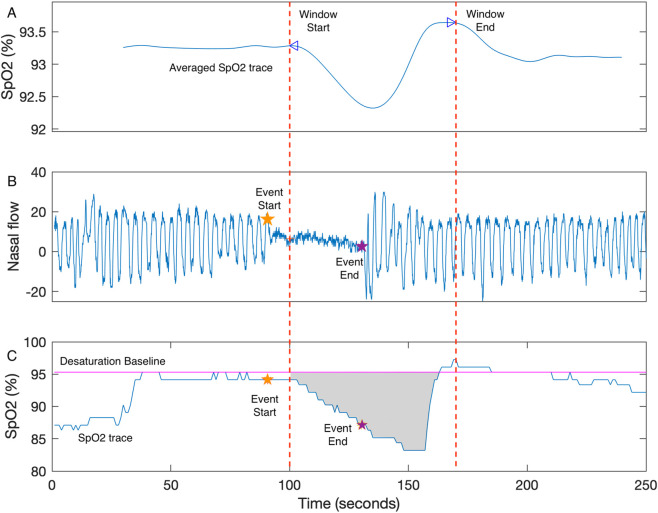
An example of hypoxic burden. **(A)** The averaged SpO2 trace (the average of the SpO2 segments associated with all respiratory events in the example recording) sets boundaries of the record-specific sampling window for hypoxic burden. **(B)** The endpoints of the example respiratory event (event start: orange star; event end: purple star) are shown on the corresponding nasal flow. **(C)** The desaturation area (shaded) of the example respiratory event is calculated as the area below desaturation baseline (magenta), above the SpO2 trace, and within the record-specific sampling window. The desaturation baseline is event-by-event based and is defined as the maximum SpO2 value within 100 s prior to the end of event (orange star) (Azarbarzin et al., 2019; He et al., 2023a). Adapted with permission from He et al. (2023a), licensed under CC BY 4.0.

**FIGURE 3:**
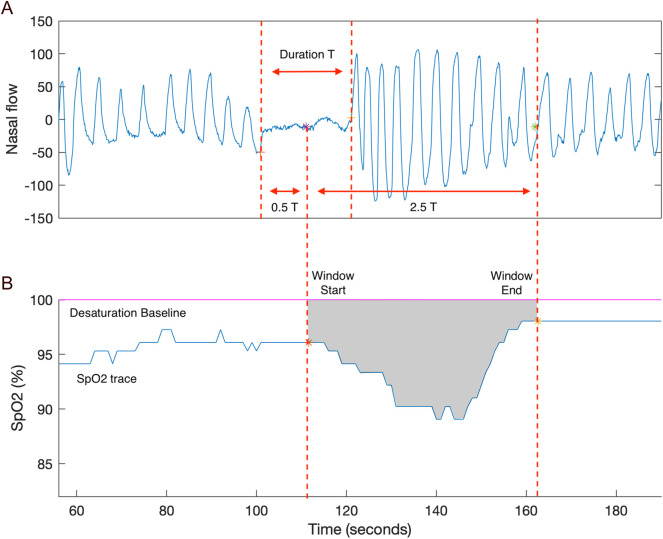
An example of REDTA. **(A)** An example of the fixed sampling window is shown on the nasal flow of a respiratory event. The fixed sampling window begins from the midway of the event and ends at 2.5 times of the event, where T is the event duration. **(B)** The desaturation area of the example respiratory event is calculated as the area within the fixed sampling window, 100% fixed baseline, and the SpO2 trace. REDTA is the sum of desaturation areas associated with scored respiratory events divided by 3,600 (de Chazal et al., 2021; He et al., 2023a). Adapted with permission from He et al. (2023a), licensed under CC BY 4.0.

**FIGURE 4:**
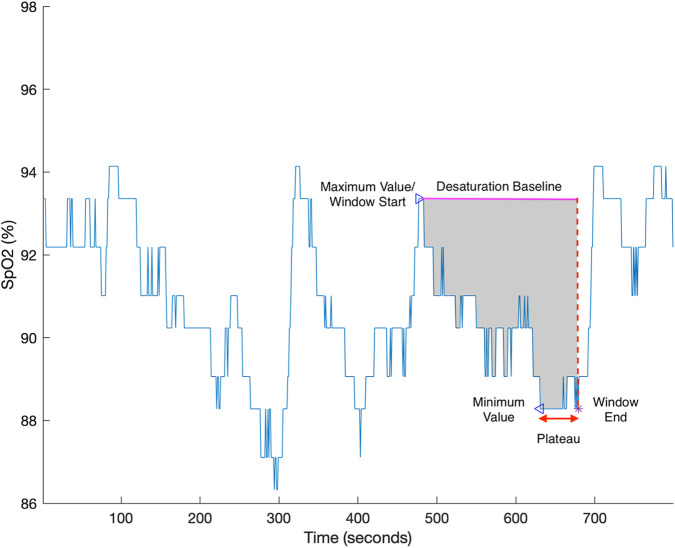
An example of desaturation severity. The desaturation area associated with an event (grey) is calculated as the area enclosed by the desaturation baseline (magenta), the SpO2 trace (blue) and the sampling window. The sampling window is demarcated by the time from the apex (maximum value; triangle) to the nadir (minimum value; triangle). When a plateau (red arrow) instead of a nadir point occurs, the end of the sampling window is shifted forward to the end of the plateau (purple star). The desaturation baseline is the maximum value within the sampling window (He et al., 2023a). Adapted with permission from He et al. (2023a), licensed under CC BY 4.0.

Desaturation area–based parameters measure the cumulative area of the oximetry trace within a sampling-window beneath a defined baseline associated with sleep events, as exampled in Figure 1. Although current published parameters share the same fundamental definition, they differ in computational approaches. In general, these discrepancies can be categorized into variations in sleep event selection, sampling-window, and baseline calculation. These differences are comparatively illustrated in Figures 2–4 (Azarbarzin et al., 2019; de Chazal et al., 2021; Kulkas et al., 2013). Each approach has demonstrated positive performance in OSA–CVD analyses but with differing levels of predictive ability for CVD outcomes (He et al., 2023a). One possible explanation is that predictive performance could be strongly influenced by computational methodology, as each approach has inherent limitations. For instance, in parameters where the baseline is tailored for each sleep event, events occurring in close succession may cause residual effects from earlier events to interfere with subsequent baseline estimation, thereby reducing the reliability of event-specific approaches (He et al., 2023b). In contrast, parameters using fixed windows and baselines adopt a “one-size-fits-all” strategy that enhances robustness but lacks adaptability to individual recordings.

To our knowledge, no study has systematically examined how computational variations affect desaturation area-based parameters or whether these differences impact their ability to capture the association between OSA and CVD. Existing studies vary in their computational methods, definitions of sleep events, and study populations, making cross-study comparisons difficult (Iber et al., 2007; Berry et al., 2012). Moreover, the current parameters have not been jointly assessed in the same database for predicting the same outcomes, thus limiting direct performance comparisons.

This study addresses these gaps by conducting a comprehensive comparison of major desaturation area-based methods within the same patient population, using consistent definitions of respiratory and desaturation events. Three algorithms were selected based on their ability to be implemented using original or validated methods: respiratory event desaturation transient area (referred as ***de Chazal method***), which was implemented as originally proposed; hypoxic burden (referred as ***Azarbarzin method***), for which a validated replication method was available; and desaturation severity (referred as ***Kulkas method***), which was supported by publicly available software (Azarbarzin et al., 2019; de Chazal et al., 2021; Kulkas et al., 2013). The aim of this study is to examine the effects of event selection, sampling-window, and baseline calculation on desaturation area-based measures for predicting CVD mortality and to identify the method that best suits this prediction.

## 2 Methods

### 2.1 Study samples

This study used data from the Sleep Heart Health Study (SHHS), a community-based multi-center cohort study conducted by the National Heart, Lung, and Blood Institute. The dataset is publicly available and was designed to investigate the consequences of sleep-disordered breathing, including cardiovascular outcomes (Dean et al., 2016; Sutherland et al., 2022). A total of 6,441 men and women aged 40 years and older participated in an unattended PSG at baseline, with a follow-up examination conducted over the subsequent decade. The follow-up collected information on CVD outcomes, demographics, smoking history, and alcohol use through interviews, self-reported questionnaires, telephone contacts, and surveillance of local hospital records and community obituaries. The CVD mortality outcomes in the SHHS were ascertained by linkage with the National Death Index, supplemented by review of death certificates and medical records, with underlying cause of death categorized using standard International Classification of Diseases codes and adjudicated by study physicians, enabling time-to-event analyses of baseline polysomnographic predictors of CVD death (Azarbarzin et al., 2019). As the SHHS dataset pre-excluded individuals with prior sleep apnea treatment, current home oxygen therapy, or tracheostomy, a total of 5,804 participants were available for this study.

As this study represents a secondary analysis of data obtained from the SHHS, no additional institutional ethics approval was required. The original study protocol was approved by the relevant institutional review boards, and ethical oversight was obtained under the SHHS coordinating body (NCT00005275).

### 2.2 Desaturation area-based methods

To achieve the goal of assessing the impact of different computational approaches on desaturation area-based parameters in predicting CVD mortality, and of identifying the method best suited for this prediction, the study assessed 15 possible combinations of desaturation area-based methods. The study can be divided into two main steps:

1. The implementation of three published base algorithms.
2. Based on the algorithm replication, 15 desaturation area-based methods were computed.

#### 2.2.1 Implementation of published algorithms

Three oximetry-derived algorithms were chosen, and all characterize the overnight desaturation area: the area between the oximetry trace and the baseline within sampling windows, which are associated with sleep events. However, they differ in computational methodology and can be categorized along three dimensions: the choice of events, the definition of sampling windows, and baselines.

The base algorithms use either manually scored respiratory events following the American Academy of Sleep Medicine (AASM) guidelines or automatically detected oxygen desaturations. The ***Azarbarzin method*** and ***de Chazal method*** are based on manually scored respiratory events, whereas ***Kulkas method*** is calculated directly from automated desaturation detection algorithms (Azarbarzin et al., 2019; de Chazal et al., 2021; Kulkas et al., 2013). The ***Kulkas method*** was chosen over other automated desaturation detection methods because it is open-sourced and supported by published software, ensuring reproducibility and alignment with the original design. Other automated algorithms, such as HBoxi, were excluded because their full implementation details have not been disclosed, limiting reproducibility and increasing the risk that differences in performance may arise from reimplementation variability rather than algorithmic differences (Esmaeili et al., 2023).

##### 2.2.1.1 *Azarbarzin* method: hypoxic burden

Hypoxic burden is a respiratory event scoring dependent algorithm that calculates the sum of the area between the SpO2 trace, and the event-specific desaturation baseline divided by the total time of sleep associated with manually score respiratory events, as shown in Equation 1 (Azarbarzin et al., 2019).

$$\text{Hypoxic burden}=\frac{\begin{array}{c} events\;\\ \sum area\;of\;an\;individual\;desaturation\;event\; \end{array}}{\sum time\;of\;sleep}$$ (1)

and the unit of hypoxic burden is %minutes per hour of sleep.

Two key components contribute to the calculation of hypoxic burden: the sampling window and the desaturation baseline. As shown in Figure 2C, the desaturation area (shaded) for a single respiratory event is defined as the area below the desaturation baseline, above the SpO2 curve and within a search window. The record-specific sampling window is indicated by the two peaks of an averaged SpO2 trace (the average of SpO2 segments associated with all respiratory events in a recording) as shown in Figure 2A. Hypoxic burden has a desaturation baseline associated with each manually scored respiratory event. The event-specific baseline is the maximum value of SpO2 signal within 100 s prior to the end of event (Figure 2C). We previously replicated the hypoxic burden algorithm, and the validated MATLAB code is publicly available in GitHub (https://github.com/pdechazal/Hypoxic-Burden).

##### 2.2.1.2 *de Chazal method:* respiratory event desaturation transient area

Respiratory event desaturation transient area (REDTA) uses a fixed window and a fixed baseline. REDTA is based on the manually scored respiratory events and calculates the sum of desaturation area between SpO2 trace and desaturation baseline associated with respiratory events divided by 3,600, as shown in Equation 2 (de Chazal et al., 2021). It measures the hypoxemia insult per night, independent of total time of sleep.

$$REDTA=\frac{\begin{array}{c} events\;\\ \sum area\;of\;an\;individual\;desaturation\;event\; \end{array}}{3600}$$ (2)

and the unit of REDTA is %hours.

REDTA has two key components in calculation: the sampling window and the baseline. As shown in Figure 3A, the fixed sampling window that starts from the respiratory event midpoint and extends for 2.5 times the event duration. The baseline is 100%. The desaturation area of an example apnea event is shown in Figure 3B. It is defined as the area bounded by the SpO2 trace, the 100% baseline and the fixed sampling window.

##### 2.2.1.3 *Kulkas method*: desaturation severity

Desaturation severity is a desaturation area-based algorithm associated with automatically detected desaturation events. Similar to the other methods, the algorithm has two key aspects: sampling window and baseline. The sampling window of desaturation severity is tailored to each event, and the duration of window does not exceed 180 s, starting from the maximum value of a candidate desaturation event and ending at the minimum value (He et al., 2023a). The desaturation baseline varies with events and is the maximum value of the desaturation event (usually the start of sampling window), as shown in Figure 4.

The desaturation severity value for a recording is calculated by summing the desaturation areas and dividing by total time of sleep. This is shown in Equation 3.

$$\text{Desaturation severity}=\frac{\sum_{n=1}^{number\;of\;desaturation\;events}{Desaturation\;area}_{n}}{\sum time\;of\;sleep}$$ (3)

and the unit of desaturation severity is % (Kulkas et al., 2013). The algorithm for calculating is desaturation severity is complex but conveniently the authors provide an online analysis package (ABOSA) that can process a supplied SpO2 signal (Karhu et al., 2022).

#### 2.2.2 Implementation of 15 desaturation area-based methods

While the implementation of the ***Azarbarzin method***, ***de Chazal method***, and ***Kulkas method*** provided the foundation, all desaturation area–based methods examined in this study were computed within the same unified framework.

These methods share a common definition: each quantifies the cumulative area where the SpO2 signal falls below a defined baseline within a sampling window associated with sleep events. Methodological variation arises from differences in event choice, sampling window definition, and baseline calculation. To ensure consistency with the published algorithms, uniform standards for event annotation, sampling windows, and baseline determination were applied across all method combinations, as summarized below.

For the events:

- **Automatically detected desaturation events** with a 3% SpO2 drop were annotated using the open-source ABOSA package, developed by the original team for calculating the ***Kulkas method*** (Kulkas et al., 2013; Karhu et al., 2022).
- **Manually scored respiratory events** were scored by the Sleep Research Centre in Boston, United States of America, in accordance with the SHHS Reading Centre Manual of Operations. Respiratory events used in this study include apnea and hypopnea events accompanied by either an oxygen desaturation ≥3% or an arousal occurring within 5 s after the event. Respiratory classification was based on airflow measurements: events with >75% reduction in airflow lasting ≥10 s were classified as obstructive apnea, while events with >30% reduction in airflow lasting ≥10 s were classified as hypopnea (Redline et al., 1998; Quan et al., 1997). The manually scored respiratory events are provided by the SHHS database.

  For the sampling windows:

- The **event-specific window** was defined as the start and end of each respiratory or desaturation event (Kulkas et al., 2013).
- The **record-specific window** was defined by the two peaks of the averaged SpO2 trace (Azarbarzin et al., 2019).
- The **fixed window** started at the midpoint of the event and extended to 2.5 times the event duration (de Chazal et al., 2021).

  For the baselines:

- The **event-specific baseline** was set as the maximum SpO2 value within 100 s before the end of the event (Azarbarzin et al., 2019), except for the ***Kulkas method***, whose baseline was calculated directly by the ABOSA software.
- The **record-specific baseline** was defined as the 99th percentile of the SpO2 signal within a single recording (He et al., 2023b).
- The **fixed baseline** was set at 100% (de Chazal et al., 2021).

While most methods were computed in MATLAB following the predefined standards above, the computation of the ***Kulkas method***, ***Kulkas method (record specific baseline*** *)*, and ***Kulkas method (fixed baseline)*** were an exception, as these were implemented using the ABOSA software. The rationale for using ABOSA for these methods, rather than manual implementation as with the others, was to ensure that observed performance differences were attributable solely to the algorithms, not to potential inconsistencies introduced during algorithm reimplementation.

  *Kulkas method*:

- Implemented directly via ABOSA software (Karhu et al., 2022).
- The event-specific baseline was defined as the maximum SpO2 value during an event (usually at the start of the event), representing a minor deviation from the definition above.

  *Kulkas method (fixed baseline)*:

- Implemented directly via ABOSA software, without deviation from the standard methodology (Karhu et al., 2022).

  *Kulkas method (record-specific baseline)*:

- Calculated using ABOSA software (Karhu et al., 2022).
- The value of the record-specific baseline was first determined. A constant value equal to the difference between this value and 100% was then added to every SpO2 sample, so that the record-specific baseline was adjusted to exactly 100%. The adjusted SpO2 signal was then processed by ABOSA, and the resulting fixed baseline output was taken as the ***Kulkas method (record-specific baseline*** *).*

Additionally, as the ABOSA software is currently unable to accept a respiratory event list, this study could not calculate three methods associated with the event-specific sampling window and manually scored respiratory events, as indicated in grey in Table 1. Consequently, a total of 15 combinations were evaluated. Although two event variations combined with three sampling window variations and three baseline variations could theoretically yield 18 combinations, three of these could not be implemented due the ABOSA software limitations.

**TABLE 1:** Summary of the desaturation area calculation methods implemented. The italicized descriptor in each table cell shows original author of the method with algorithm modifications shown in brackets.

​	Manually scored respiratory events			Automatically detected desaturation events		
Sampling-window	Event-specific baseline	Record-specific baseline	Fixed baseline	Event-specific baseline	Record-specific baseline	Fixed baseline
Event-specific	​	​	​	*Kulkas method*	*Kulkas method (record-specific baseline)*	*Kulkas method (fixed baseline)*
Record-specific	*Azarbarzin method*	*Azarbarzin method (record- specific baseline)*	*Azarbarzin method (fixed baseline)*	*Azarbarzin method (desaturation events)*	*Azarbarzin method (record-specific baseline, desaturation events)*	*Azarbarzin method (fixed baseline, desaturation events)*
Fixed	*de Chazal method (event-specific baseline)*	*de Chazal method (record-specific baseline)*	*de Chazal method*	*de Chazal method (event-specific baseline, desaturation events)*	*de Chazal method (record-specific baseline, desaturation events)*	*de Chazal method (desaturation events)*

Due to limitations of software implementing ***the Kulkas method***, we were unable to implement the three methods shaded grey.

### 2.3 Statistical analysis

The results of each desaturation area method were standardized (z-scores) and treated as distinct SpO2 predictors of CVD mortality (Sutherland et al., 2022). Standardization was necessary to allow direct comparison across parameters with different units. It was performed by subtracting the mean value of each set of desaturation areas and dividing by its corresponding standard deviation. Hazard ratio (HR), p-values, and associated 95% confidence intervals (95% CI), derived from the Cox proportional hazards regression analysis (Cox and Oakes, 1984), were used to compare the ability of each desaturation area method to predict CVD mortality. A p-value threshold of 0.05 was applied to determine statistical significance, and a threshold of 0.10 was applied to indicate statistical trend. Due to the high correlation of our calculated desaturation area measures and as it was an exploratory study, we did not apply a significance correction (such as Bonferroni correction) to our p-values. A higher hazard ratio with statistical significance indicated a stronger predictive ability of the desaturation area method for CVD mortality.

The Cox proportional hazards regression models in this study were adjusted using the same covariates as those in Model 4 of the study by Azarbarzin et al. (Azarbarzin et al., 2019). These covariates included demographic variables (age, race, gender, and total sleep time), smoking status, alcohol use, chronic obstructive pulmonary disease (COPD) history, AHI, Time below 90% oxygen saturation (T90), event-related minimum oxygen desaturation (MinSat), and concurrent cardio-metabolic diseases (heart failure, stroke, angina, coronary revascularization, and myocardial infarction).

## 3 Results

### 3.1 Sample selection and characteristics

**FIGURE 5:**
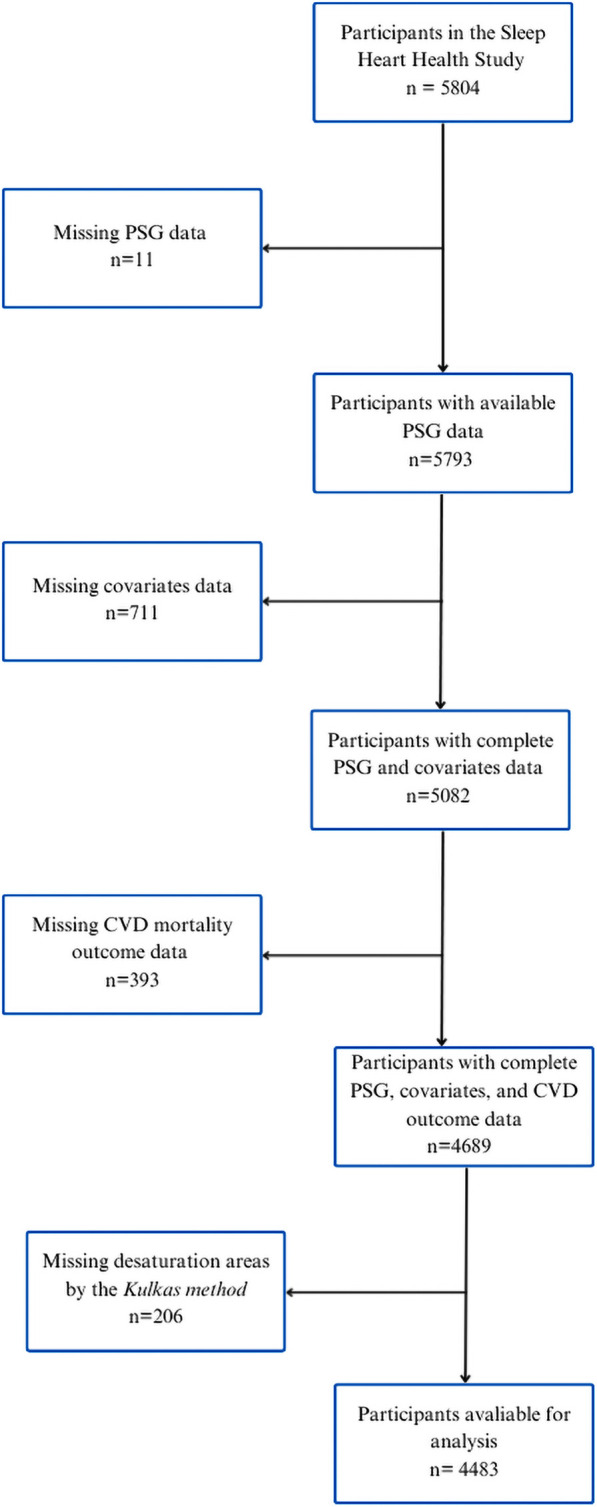
Flowchart of study sample selection from the SHHS cohort, with additional exclusion of participants missing desaturation area values (***Kulkas method***) to ensure consistency across methods.

Of the 5,804 participants who completed the study, 11 were excluded due to missing PSG data, 711 due to missing covariate data, 393 due to missing CVD mortality outcome data and 206 were unable to be successfully processed by the ***Kulkas method***. This resulted in a final sample of 4,483 participants eligible for analysis, as depicted in Figure 5. Within this cohort, there were 311 CVD deaths (Putcha et al., 2016). The sample characteristics are presented in Table 2. In the group of participants who remained free from CVD mortality (CVD survivor group), females comprised 53.91% of participants, whereas in the CVD mortality group, males were more prevalent, accounting for 53.38%. Participants were predominantly Caucasian in both the survivor group (88.49%) and the mortality group (88.75%). The mean age was 63.47 years in the survivor group and 75.79 years in the mortality group. The mean AHI indicated moderate OSA, with values of 17.73 in the survivor group and 20.94 in the mortality group.

**TABLE 2:** Sample characteristics of the SHHS involved in the analysis.

Variables	Total n = 4483 (100%)	
	CVD survivor n = 4172 (93.1%)	CVD death N = 311 (6.9%)
Age (years), mean (SD)	63.47 (10.64)	75.79 (7.60)
BMI (kg/m^2^), mean (SD)	28.34 (5.09)	27.33 (4.79)
Race		
Caucasian, n (%)	3692 (88.49)	276 (88.75)
Other, n (%)	480 (11.51)	35 (11.25)
Gender		
Male, n (%)	1923 (46.09)	166 (53.38)
Female, n (%)	2249 (53.91)	145 (46.62)
Smoking status		
Never, n (%)	1941 (46.53)	142 (45.66)
Former, n (%)	1830 (43.86)	148 (47.59)
Current, n (%)	401 (9.61)	21 (6.75)
Total time of sleep (TST), n (%)		
≤5 h	626 (15.00)	79 (25.40)
5–8 h	3499 (83.87)	230 (73.95)
≥8 h	47 (1.13)	2 (0.65)
T90 (%TST), mean (SD)	3.36 (9.96)	6.01 (15.53)
AHI (events/h), mean (SD)	17.73 (15.73)	20.94 (16.00)
COPD, n (%)	50 (1.20)	3 (0.96)
Stroke, n (%)	125 (3.00)	32 (10.29)
Heart failure, n (%)	57 (1.37)	21 (6.75)
Diabetes, n (%)	254 (6.09)	65 (20.90)
Hypertension, n (%)	1576 (37.78)	218 (70.10)
Lipid-lowering medication use, n (%)	507 (12.15)	50 (16.08)

### 3.2 Comparison of desaturation area-based parameters in predicting CVD mortality

To evaluate the predictive efficacy of fifteen desaturation area-based methods, Table 3 and 4 present the adjusted HRs and corresponding 95% CI for normalized desaturation areas as predictors of CVD mortality. Table 3 provides HRs for the partially adjusted model with covariate adjustments for demographic factors, smoking status, alcohol intake, and COPD history. Table 4 provides HRs for the fully adjusted model with covariate adjustments for all Table 3 covariates, AHI, T90, MinSat, and concurrent cardio-metabolic diseases.

**TABLE 3:** Desaturation area-based methods predicting CVD mortality with partially adjusted model.

​	Manually scored respiratory events			Automatically detected desaturation events		
Sampling-window	Event-specific baseline	Record-specific baseline	Fixed baseline	Event-specific baseline	Record-specific baseline	Fixed baseline
Event-specific	​	​	​	1.06 (0.89–1.27)	1.10 (0.92–1.31)	1.08 (0.90–1.29)
Record-specific	1.34 (0.96–1.87) p = 0.09	1.57 (1.09–2.27)p = 0.02	1.62 (1.08–2.42) p = 0.02	1.03 (0.87–1.23)	1.06 (0.89–1.27)	1.05 (0.88–1.26)
Fixed	1.19 (0.94–1.51)	1.30 (1.00–1.69)p = 0.04	1.31 (0.99–1.73) p = 0.06	1.00 (0.88–1.14)	1.03 (0.89–1.18)	1.01 (0.89–1.16)

The hazard ratios and corresponding 95% confidence intervals are shown for evaluating the performance. P-values shown when less than 0.1.

**TABLE 4:** Desaturation area-based methods predicting CVD mortality with fully adjusted model.

​	Manually scored respiratory events			Automatically detected desaturation events		
Sampling-window	Event-specific baseline	Record-specific baseline	Fixed baseline	Event-specific baseline	Record-specific baseline	Fixed baseline
Event-specific	​	​	​	1.02 (0.79–1.32)	1.08 (0.84–1.39)	1.02 (0.79–1.32)
Record-specific	1.56 (0.84–2.89)	1.79 (1.00–3.19)p = 0.04	1.69 (0.91–3.15) p = 0.1	0.96 (0.74–1.23)	1.00 (0.78–1.28)	0.98 (0.76–1.25)
Fixed	1.24 (0.79–1.96)	1.53 (0.93–2.52)p = 0.09	1.47 (0.88–2.46)	0.94 (0.82–1.07)	0.96 (0.83–1.11)	0.95 (0.82–1.09)

The hazard ratios and corresponding 95% confidence intervals are shown for evaluating the performance. P-values are shown when less than 0.1.

The results in Table 3 and 4 indicate that the desaturation areas based on automated automatically detected desaturation events were unsuccessful in predicting CVD mortality as all p-values of the HRs were greater then 0.1, for partially and fully adjusted covariate models. In contrast, the p-values of the HRs for the desaturation areas based on manually scored respiratory events were less than 0.1 for the partially adjusted model (Table 3) suggesting a trend of this group of methods providing independent predictive performance. The ***Azarbarzin method (record-specific baseline)***, ***Azarbarzin method (fixed baseline)***, ***and de Chazal method (record-specific baseline)*** models all demonstrated statistical significance. These results did not hold up for the fully adjusted model (Table 4). The only parameter that achieved statistical significance was ***Azarbarzin method (record-specific baseline)***, (HR of 1.79 (95% CI: 1.00–3.19)). ***Azarbarzin method (fixed baseline)***, and ***de Chazal method (record-specific baseline)*** demonstrated a statistical trend with a p-value less than 0.1 and all other parameters resulted in a p-value greater 0.1.

## 4 Discussion

This study conducted a comprehensive comparison of desaturation area-based methods to evaluate their effectiveness in predicting CVD mortality among middle-aged and older adult cohorts. Variations in event choice, sampling-window, and baseline were examined, and the predictive performance of each was assessed. Overall, parameters linked to manually scored respiratory events demonstrated some evidence of predictive ability for CVD mortality in a community-based cohort. Among these, methods using record-specific sampling-windows achieved the strongest performance in both partially and fully adjusted models. In the fully adjusted model, the ***Azarbarzin method (record-specific baseline)*** outperformed all others with a significant hazard ratio. The ***Azarbarzin method (fixed baseline)*** and ***de Chazal method (record-specific baseline)*** were significant in the partially adjusted model but became marginally significant after adjustment for all covariates. By contrast, parameters derived from automatically detected desaturation events showed minimal evidence of predictive value. The automated desaturation–based methods yielded p-values greater than 0.1 across all levels of covariate adjustment, indicating negligible predictive ability.

With the goal of identifying a method that reliably predicts CVD mortality, the ***Azarbarzin method (record-baseline)*** emerges as the most promising approach. Its record-specific sampling-window is tailored to each recording, reducing the risk of incomplete capturing of prolonged desaturation events. The record-specific baseline offers greater noise tolerance than event-specific baselines, minimizing the effects of recording quality. The ***Azarbarzin method (record-specific baseline)*** therefore strikes an optimal balance between individualized analysis and noise resistance, showing strong potential for CVD mortality prediction. This method represents a meaningful step towards early risk stratification in CVD patients.

Beyond predictive performance, this study addressed the reasons underlying the differences between methods based on manually scored respiratory events and those based on automatically detected desaturation events. As shown in Table 3 and 4, methods triggered by automated desaturation events yielded insignificant HR values across both models, suggesting no predictive ability for CVD mortality. By contrast, methods with the same paired sampling-windows and baseline choices but based on manually scored respiratory events demonstrated promising outcomes. For example, the ***de Chazal method (record-specific baseline)*** and ***de Chazal method (record-specific baseline, desaturation events)*** shared the same fixed sampling-window and record-specific baseline yet produced markedly different outcomes: the former achieving a significant HR (1.53), whereas the latter showing an insignificant HR (0.96) in the fully adjusted models. The only difference between these methods was the choice of events. This indicates that event definition strongly influences the predictive ability of desaturation area-based methods.

**FIGURE 6:**
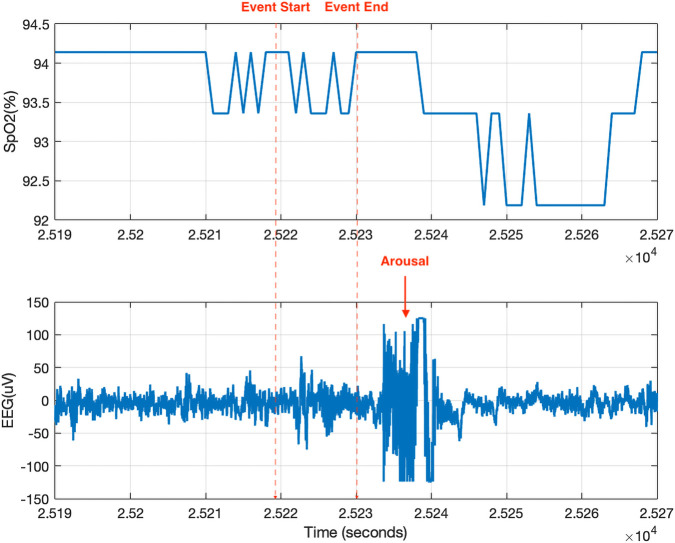
An example of a hypopnea event accompanied by an arousal event (identified based on EEG signal) that begins within 5 s after the hypopnea event ends. If the associated desaturation is less than 3% (as illustrated in this figure), such events can only be identified through manual respiratory event scoring, which relies on expert input and multiple signals from overnight PSG. Consequently, the number of manually scored respiratory events is typically higher than that of automatically detected desaturation events.

A possible explanation for this inconsistency is that automated desaturation detection neglects arousal-related events. Automated events were derived solely from oximetry by scanning for desaturations ≥3%. In contrast, manually scored respiratory events are annotated according to AASM scoring guidelines, incorporating both desaturations and arousals. Because of this difference in annotation, arousal-related hypopnea events may be undercounted by automated detection algorithms. As exampled in Figure 6, a hypopnea event scored in the SHHS database was associated with an arousal that occurred within 5 s after the event ended, despite the absence of a desaturation greater than 3%. Such events are included in area calculations when respiratory scoring is used but excluded by automated desaturation-based algorithms. In the SHHS recording, manually scored respiratory events outnumbered automated desaturation events by an average factor of five, suggesting that a substantial number of clinically relevant events are omitted by automated approaches using a 3% threshold.

Esmaeili et al. similarly reported that the automated HBoxi algorithm, using a 2% threshold, predicted CVD outcomes, but applying the AASM-recommended 3% threshold eliminated statistical significance (Esmaeili et al., 2023). This suggests that automated methods using thresholds aligned with AASM criteria may not provide a valid characterization of hypoxia, thereby reducing their predictive performance. Although HBoxi could not be included in this study due to unavailable implementation details, their findings align with the present results: desaturation area-based methods are highly sensitive to event definition. Even when the sampling-window and baseline are identical, the definition of annotated sleep events can substantially influence the predictive performance. Therefore, future development of automated desaturation area-based methods must carefully consider event annotation strategies to ensure accurate characterization of overnight hypoxia.

Notwithstanding the strengths of this study, there are some important limitations. The SHHS represents a community-based cohort of middle-aged and older adults, predominantly Caucasian, with limited information on OSA treatment history. As such, the cohort may not fully reflect the increasingly younger and more diverse population currently seen in contemporary sleep clinics. Younger patients may present with prolonged flow limitation that can evolve into more overt respiratory events, posing challenges in accurately determining event onset. The duration of OSA in patients is unknown, which may influence the cardiovascular adaptation to long-term hypoxemia and hence affect the performance of oximetry-derived desaturation area-based parameters in predicting CVD outcomes (Redline et al., 1998). In addition, airflow in SHHS was measured using thermistors, which are less sensitive than nasal pressure transducers and may influence the sleep event annotation.

Nevertheless, SHHS remains appropriate for the present aims, as evaluation of CVD mortality requires extended (>10 years) follow-up with CVD outcomes, which provides well-characterized polysomnographic data. Besides, although thermistors were used for airflow measurement, the desaturation area parameters evaluated in this study are derived from oximetry signals rather than airflow signals. Respiratory events in SHHS were scored according to the prevailing AASM guidelines, and the cohort has been extensively utilized in OSA–CVD research, supporting its validity for long-term CVD outcome analysis despite these constraints. In light of these limitations, future studies should seek to validate the present findings in larger, more diverse, and younger clinical cohorts, including datasets composed exclusively of patients with diagnosed OSA. Additionally, replication in databases utilizing nasal pressure–based airflow measurements would enable more precise event characterization and further strengthen risk prediction analysis.

Furthermore, we made comparisons between manually scored respiratory events and automatically detected desaturation events were conducted in one database. To derive more reliable conclusions regarding the impact of automated desaturation event detection on CVD mortality prediction, further investigations using multiple databases are necessary. Additionally, the automatic detection algorithm used in this study is only one of several available methods and does not represent the performance of all published algorithms. Apart from HBoxi discussed above (Esmaeili et al., 2023), Terrill et al. introduced an algorithm for detecting desaturation events and calculating a dynamic baseline for oximetry data (Terrill et al., 2018). Both approaches could be explored in future research.

Finally, each desaturation area–based approach has inherent methodological limitations. Event-specific baseline methods may be more sensitive to signal noise, whereas fixed-baseline approaches may impose a “one-size-fits-all” assumption that overlooks inter-individual variability. Although the present study systematically evaluated 15 possible combinations, the analysis was restricted to currently published algorithms. Certain physiological scenarios may therefore remain insufficiently captured by existing methods. For example, progressive reductions in oxygen saturation without clear renaturation to baseline, which is commonly observed in severe disease or during REM sleep. Hypoventilation, a persistently low baseline with only minor event-related fluctuations may lead to substantial time spent below thresholds (e.g., 90% or 88%). Overall, current approaches are primarily designed to quantify general desaturation burden rather than to target specific physiological scenarios. While this is a common and practical strategy when developing algorithms for large-scale databases, it may overlook atypical or clinically nuanced patterns. It would be interesting to systematically examine the impact of these specific scenarios on desaturation area metrics and their association with CVD outcomes. Future research should focus on methodological refinement to better characterize these unresolved physiological patterns and to determine whether tailored metrics can improve CVD risk prediction in specific clinical phenotypes.

## 5 Conclusion

We explored different baseline calculation methods for desaturation area-based parameters and conducted a comprehensive comparison of their predictive performance for CVD mortality. The findings indicate that the choice of event annotations significantly influences the predictive performance of desaturation area-based parameters. Among the evaluated approaches, the ***Azarbarzin method (record-specific baseline)*** demonstrated the strongest predictive performance. This method utilizes a **record-specific sampling-window** and **baseline**, effectively minimizing noise interference. We believe that this approach represents a step toward identifying oximetry parameters that enable early risk stratification in CVD patients.

## Data Availability

Publicly available datasets were analyzed in this study. This data can be found here: https://biolincc.nhlbi.nih.gov/studies/shhs/.
